# Supplementary material for: Pyruvate Dehydrogenase Kinase 1 inhibition mediated oxidative phosphorylation enhancement in cartilage promotes osteoarthritis progression
Source: BMC Musculoskelet Disord. 2023 Jul 20;24:597. doi: 10.1186/s12891-023-06585-6 (PMC10357736; doi:10.1186/s12891-023-06585-6)
Supplement: Supplementary file 1 — Additional file 1. [file 12891_2023_6585_MOESM1_ESM.zip › MF.Gsea.1653627316242/GOMF_AROMATASE_ACTIVITY.html]

Details for gene set GOMF\_AROMATASE\_ACTIVITY[GSEA]

|  || Dataset | 55457\_collapsed\_to\_symbols.cls.cls #OA\_versus\_normal.cls.cls #OA\_versus\_normal\_repos |
| Phenotype | cls.cls#OA\_versus\_normal\_repos |
| Upregulated in class | normal |
| GeneSet | GOMF\_AROMATASE\_ACTIVITY |
| Enrichment Score (ES) | -0.56602955 |
| Normalized Enrichment Score (NES) | -1.8088354 |
| Nominal p-value | 0.0035778175 |
| FDR q-value | 0.083715685 |
| FWER p-Value | 0.508 |
Table: GSEA Results Summary

  

Fig 1: Enrichment plot: GOMF\_AROMATASE\_ACTIVITY      
 Profile of the Running ES Score & Positions of GeneSet Members on the Rank Ordered List

  

| SYMBOL | TITLE | RANK IN GENE LIST | RANK METRIC SCORE | RUNNING ES | CORE ENRICHMENT || 1 | CYP3A43 | cytochrome P450 family 3 subfamily A member 43 [Source:HGNC Symbol;Acc:HGNC:17450] | 4698 | 0.096 | -0.3479 | No |
| 2 | CYP4F11 | cytochrome P450 family 4 subfamily F member 11 [Source:HGNC Symbol;Acc:HGNC:13265] | 5144 | 0.060 | -0.3719 | No |
| 3 | CYP4F8 | cytochrome P450 family 4 subfamily F member 8 [Source:HGNC Symbol;Acc:HGNC:2648] | 5326 | 0.046 | -0.3779 | No |
| 4 | CYP1B1 | cytochrome P450 family 1 subfamily B member 1 [Source:HGNC Symbol;Acc:HGNC:2597] | 5925 | 0.000 | -0.4242 | No |
| 5 | CYP2C19 | cytochrome P450 family 2 subfamily C member 19 [Source:HGNC Symbol;Acc:HGNC:2621] | 6089 | -0.011 | -0.4349 | No |
| 6 | CYP2C18 | cytochrome P450 family 2 subfamily C member 18 [Source:HGNC Symbol;Acc:HGNC:2620] | 6210 | -0.022 | -0.4404 | No |
| 7 | CYP19A1 | cytochrome P450 family 19 subfamily A member 1 [Source:HGNC Symbol;Acc:HGNC:2594] | 6388 | -0.034 | -0.4482 | No |
| 8 | CYP2F1 | cytochrome P450 family 2 subfamily F member 1 [Source:HGNC Symbol;Acc:HGNC:2632] | 7907 | -0.148 | -0.5400 | Yes |
| 9 | CYP1A1 | cytochrome P450 family 1 subfamily A member 1 [Source:HGNC Symbol;Acc:HGNC:2595] | 8195 | -0.171 | -0.5323 | Yes |
| 10 | CYP3A5 | cytochrome P450 family 3 subfamily A member 5 [Source:HGNC Symbol;Acc:HGNC:2638] | 8272 | -0.177 | -0.5071 | Yes |
| 11 | CYP2C9 | cytochrome P450 family 2 subfamily C member 9 [Source:HGNC Symbol;Acc:HGNC:2623] | 8595 | -0.201 | -0.4968 | Yes |
| 12 | CYP4A11 | cytochrome P450 family 4 subfamily A member 11 [Source:HGNC Symbol;Acc:HGNC:2642] | 8654 | -0.205 | -0.4653 | Yes |
| 13 | CYP3A7 | cytochrome P450 family 3 subfamily A member 7 [Source:HGNC Symbol;Acc:HGNC:2640] | 9024 | -0.234 | -0.4528 | Yes |
| 14 | CYP4F3 | cytochrome P450 family 4 subfamily F member 3 [Source:HGNC Symbol;Acc:HGNC:2646] | 9224 | -0.250 | -0.4242 | Yes |
| 15 | CYP4F2 | cytochrome P450 family 4 subfamily F member 2 [Source:HGNC Symbol;Acc:HGNC:2645] | 9599 | -0.283 | -0.4034 | Yes |
| 16 | CYP4B1 | cytochrome P450 family 4 subfamily B member 1 [Source:HGNC Symbol;Acc:HGNC:2644] | 9670 | -0.289 | -0.3580 | Yes |
| 17 | CYP3A4 | cytochrome P450 family 3 subfamily A member 4 [Source:HGNC Symbol;Acc:HGNC:2637] | 10193 | -0.340 | -0.3388 | Yes |
| 18 | CYP2A13 | cytochrome P450 family 2 subfamily A member 13 [Source:HGNC Symbol;Acc:HGNC:2608] | 10732 | -0.397 | -0.3108 | Yes |
| 19 | CYP2E1 | cytochrome P450 family 2 subfamily E member 1 [Source:HGNC Symbol;Acc:HGNC:2631] | 10988 | -0.428 | -0.2552 | Yes |
| 20 | CYP4F12 | cytochrome P450 family 4 subfamily F member 12 [Source:HGNC Symbol;Acc:HGNC:18857] | 11423 | -0.490 | -0.2027 | Yes |
| 21 | CYP1A2 | cytochrome P450 family 1 subfamily A member 2 [Source:HGNC Symbol;Acc:HGNC:2596] | 11719 | -0.536 | -0.1313 | Yes |
| 22 | CYP2C8 | cytochrome P450 family 2 subfamily C member 8 [Source:HGNC Symbol;Acc:HGNC:2622] | 11958 | -0.585 | -0.0468 | Yes |
| 23 | CYP2D7 | cytochrome P450 family 2 subfamily D member 7 (gene/pseudogene) [Source:HGNC Symbol;Acc:HGNC:2624] | 12317 | -0.681 | 0.0453 | Yes |
Table: GSEA details [plain text format]

  

Fig 2: GOMF\_AROMATASE\_ACTIVITY      
 Blue-Pink O' Gram in the Space of the Analyzed GeneSet

  

Fig 3: GOMF\_AROMATASE\_ACTIVITY: Random ES distribution      
 Gene set null distribution of ES for **GOMF\_AROMATASE\_ACTIVITY**
